# A New Strategy to Produce a Defensin: Stable Production of Mutated NP-1 in Nitrate Reductase-Deficient *Chlorella ellipsoidea*

**DOI:** 10.1371/journal.pone.0054966

**Published:** 2013-01-28

**Authors:** Li-Li Bai, Wei-Bo Yin, Yu-Hong Chen, Li-Li Niu, Yong-Ru Sun, Shi-Min Zhao, Fu-Quan Yang, Richard R.-C. Wang, Qing Wu, Xiang-Qi Zhang, Zan-Min Hu

**Affiliations:** 1 Institute of Genetics and Developmental Biology, Chinese Academy of Sciences, Beijing, People’s Republic of China; 2 Laboratory of Proteomics, Institute of Biophysics, Chinese Academy of Sciences, Beijing, People’s Republic of China; 3 USDA-ARS, FRRL, Utah State University, Logan, Utah, United States of America; 4 Polo Biology Science Park Co., Ltd., Beijing, People’s Republic of China; Center for Genomic Regulation, Spain

## Abstract

Defensins are small cationic peptides that could be used as the potential substitute for antibiotics. However, there is no efficient method for producing defensins. In this study, we developed a new strategy to produce defensin in nitrate reductase (NR)-deficient *C. ellipsoidea* (*nrm-*4). We constructed a plant expression vector carrying mutated *NP*-1 gene (*mNP*-1), a mature α-defensin *NP*-1 gene from rabbit with an additional initiator codon in the 5′-terminus, in which the selection markers were *Npt*II and *NR* genes. We transferred *mNP*-1 into *nrm-*4 using electroporation and obtained many transgenic lines with high efficiency under selection chemicals G418 and NaNO_3_. The mNP-1 was characterized using N-terminal sequencing after being isolated from transgenic lines. Excitingly, mNP-1 was produced at high levels (approximately 11.42 mg/l) even after 15 generations of continuous fermentation. In addition, mNP-1 had strong activity against *Escherichia coli* at 5 µg/ml. This research developed a new method for producing defensins using genetic engineering.

## Introduction

Defensins, a kind of small cationic peptides with 18–54 amino acids including four or six conserved cysteine residues forming intra-molecular disulfide bonds**,** constitute the first line of defence against pathogens for innate immune system in animals and human. These peptides not only display a broad spectrum of anti-microbial activity against gram-positive and gram-negative bacteria, yeasts, fungi, enveloped viruses, parasites and protozoa [1], but also modulate the immune responses in mammals, such as enhancing phagocytosis, stimulating prostaglandin release, promoting recruitment and accumulation of various immune cells at inflammatory sites, promoting angiogenesis, influencing dendritic cell development, and inducing wound repair [2]. Mammalian defensins are divided into α-, β- and θ-defensins on the basis of the position of their disulphide bonds, their sequences and structural features [3]. Their molecular mechanism of action involves non-specific membrane permeation and disruption; thus, defensins can kill multi-drug-resistant bacteria without inducing bacterial mutations that cause antibiotic resistance [4]. Accordingly, they could be used as a new generation of antimicrobials that have a broad range of application for the topical and systemic treatment of infections [5].

However, one of the major limitations to the use of defensins is the lack of efficient production protocols [4]. There are two methods of producing defensins. One method involves their extractions from the immune cells, such as neutrophils, epithelial cells, certain macrophage populations and paneth cells of the small intestine [6]; this technique is inefficient due to limited available source. The other method involves the chemical synthesis of the peptides, which also results in low yields at high costs because it requires additional in vitro protein folding steps to form appropriate disulfide bonds [7]. Currently, genetic engineering has become the main mechanism by which large amounts of pharmaceutical proteins are generated. It should also be useful for the large-scale production of defensins. Different expression systems have been studied and developed, such as *Escherichia coli*, yeasts and plant cells [8].

Some reports have indicated that *E*. *coli* express large quantity of defensins as inclusion or fusion protein, however, the quantity of active defensins is low [8]. Yeast can express and secret active human β-defensin HBD-1. However, with increased culture time, the content in liquid medium decreased from 55 µg/l at 48 h to 9 µg/l at 120 h due to the low stability of HBD-1 at 28°C and the strong proteolytic activity in the fermentation medium [9]. Recently, *E. coli* cell-free expression systems were used to produce defensins and resulted in up to 2.0 mg/ml HBD-2 [10]. However, the development of this expression system is still being conducted.

The mature rabbit neutrophils peptide-1 (NP-1), an α-defensin, comprises 33 amino acid residues and is rich in arginines and cysteines that form three intra-molecular disulfide bonds. It has a broad spectrum of anti-microbial activity against bacterial (*Streptococcus faecalis*, *Bacillus subtilis*, *Salmonella typhimurium*, *Staphylococcus aureus*, *Staphylococcus epidermidis*, *Streptococcus pneumonia*, *Streptococcus agalactiae*, *Listeria monocytogenes*, *Pseudomonas aeruginosa*, *Escherichia coli*, *Klebsiella pneumonia*, *Serratia marcescens*, *Haemophilus influenza*, *Eikenella corrodens*, *Capnocytophaga* spp) [10]–[12], fungi (*Candida albicans*, *Aspergillus fumigatus*, *Rhizopusoryzae*) [13], virus (Avian influenza virus, herpes simplex virus type 1 and 2) [14], *Actinobacillus actinomycetemcomitans* [12], and *Treponema pallidum* [15]. It also lysed tumor cell in vitro [16], induced wound repair [17], restored the functions of the injured nerve trunk [18], and secreted histamine from mast cells at nanomolar concentrations [19]. The over-expression of NP-1 in plants enhanced resistance of transgenic higher plants against bacterial wilt, fungal attack and viral infection [20].

*Chlorella* is a kind of unicellular algae that can be used as feed source; it grows rapidly under heterotrophic culture, and its culture cost is relatively low [21]. These characteristics make it suitable for producing valuable products using genetic engineering. However, research on genetic engineering in *Chlorella* is limited. Transient expression of foreign genes has been achieved previously in *Chlorella* [22]–[23]. Human growth hormone (HGH) was expressed transiently in *C. vulgaris* C-27 and *C. sorokiniana*; however, none of the resulting colonies produced HGH [24]. *C. ellipsoidea* protoplasts were used to express the flounder growth hormone (FGH), whose expression level was estimated at 400 µg/l by ELISA [25]. We previously used *Chlorella* to successfully express NP-1 [26], but did not obtain lines that stably expressed NP-1 because the NP-1-expressing cells were undetectable when the selection chemical G418 was removed from the medium (unpublished data).

Here, we transformed a nitrate reductase (NR)-deficient *C. ellipsoidea*, strain *nrm*-4 with mutated *NP*-1 (*mNP*-1) that contained mature *NP*-1 sequence with an additional initiator codon in the 5′-terminal; we obtained lines that stably produced mNP-1 at high yield (11.42 mg/l) for 15 generation of continuous fermentation without selection chemicals. It also exhibited robust anti-microbial activity. Our research opens a new door for large-scale production of defensins.

## Materials and Methods

### Strains and Culture Conditions

NR-deficient *C. ellipsoidea* mutant *nrm*-4 [27] was used as the host cells. The stock culture of *nrm*-4 was maintained on an E4 agar slant medium containing SE (Brostol’s solution) [28] with modification, in which NaNO_3_ was substituted with 0.7 g/l NaNO_2_ and 15 g/l agar. The cells were preliminarily grown in a flask containing 20 ml of E4 solution on a rotary shaker (120 rpm) at 22°C under continuous illumination at 60 µmol/m^2^/s with a 16 h light/8 h dark cycle for 7 days. These cells were transferred to another flask containing 100 ml of E4 solution and cultured on a rotary shaker (160 rpm) at 22°C under continuous illumination (156 µmol/m^2^/s) with a 16 h light/8 h dark cycle. After 4 days, *nrm-*4 cells were harvested by centrifugation and used as host cells.

### Construction of the Plant Expression Vector

**Figure 1:**
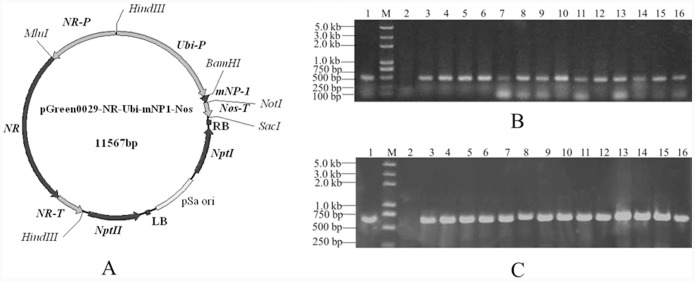
mNP-1 transformation and transgenic strain detection. ( A**)** A schematic map of the pGreen0029-NR-Ubi-mNP1-Nos plasmid. The pGreen0029-NR-Ubi-mNP1-Nos vector contained: an expression box of the NR gene from *Chlorella ellipsoidea* under the control of its own promoter (*NR*-P) and terminating in the 3′-UTR (*NR*-T) of the NR gene; an expression box of the mNP-1 gene (the mature NP-1 sequence with an additional Met at the N-terminus), controlled by the Ubiquitin gene-1 promoter (*Ubi*-P) and terminated by the Nos terminator (*Nos*-T); and an expression box of the neomycin phosphotransferase enzyme (NptII), which conferred resistance to aminoglycoside antibiotics. **(**B**)** PCR detection of the *Npt*II gene using primers 1 and 2 (Table 1) and (C) identification of Ubi-mNP1-Nos fragments using primers 3 and 4 (Table 1) for transgenic *nrm-4* cells. (1) pGreen0029-NR-Ubi-mNP1-Nos plasmid, (M) DNA molecular weight marker; (2) untransformed *nrm*-4 cells; (3–16) strains of transgenic *nrm*-4 cells.

Two plasmids pSoup and pGreen0029-NR-Ubi-mNP1-Nos (Fig. 1A) containing two selectable markers were used to transform *nrm*-4. The pSoup plasmid is a co-transfected plasmid (Biotechnology and Biological Sciences Research Council, BBSRC, UK) that aids the fragment in pGreen0029-NR-Ubi-mNP1-nos between LB and RB integrate into the genome of *nrm*-4. The vector pGreen0029-NR-Ubi-mNP1-Nos contained an expression box of NR gene from *C. ellipsoidea* (GenBank NO. AY275834) under the control of its own promoter (GenBank NO. AY307383, named *NR*-P in vector) and terminated with the 3′-UTR (untranslated region, named *NR*-T in vector) of NR gene; an expression box of mNP-1 gene (i.e., mature NP-1 sequence with an additional initiator codon in the 5′-end), which was under the control of Ubiquitin (Ubi1) gene promoter from maize [29] and terminated with the Nos terminator; and the expression box of neomycin phosphotransferase enzyme (NptII), which conferred resistance to aminoglycoside antibiotics, such as G418 (geneticin). During the construction of this vector, *mNP*-1 gene, *Nos* terminator, and *Ubiquitin* (*Ubi1*) gene promoter with designed appropriate digestion sites were digested with following double enzymes *BamH*I-*Not*I, *Not*I-*Sac*I and *Hind*III-*BamH*I, respectively. And they were subsequently ligated into the vector pGreen 0029 (BBSRC, UK) digested with the same enzymes. Finally, the *NR* expression box was inserted into the vector pGreen 0029, using *Hind*III, to complete the structure of pGreen0029-NR-Ubi-mNP1-Nos (Fig. 1A).

### Transformation of NR-deficient Chlorella Ellipsoidea Mutant

The *nrm*-4 cells were cultured to logarithmic phase, harvested following centrifugation at 50 ×g for 10 min and then processed with high-exudate buffer containing 0.2 M sorbitol and mannitol on ice for 1.5 h. The processed cells were then harvested following centrifugation at 50 ×g for 10 min, re-suspended with a buffer containing 0.2 M sorbitol, 0.2 M mannitol, 0.08 M KCl, 0.005 M CaCl_2_ and 0.01 M Hepes at a concentration of approximately 1 ×10^5^ cells/ml and then immediately mixed with a final concentration of 20 µg/ml plasmid pGreen0029-NR-Ubi-mNP1-Nos, and a final concentration of 10 µg/ml plasmid pSoup and 25 µg/ml salmon sperm DNA. The mixture was placed on ice for 10 min, and then 200–400 µl mixture was removed for transformation with a Baekon 2000 (Baekon Co., CA, USA) electroporation device. The cells were transformed using 6 KV, 0.001–0.2 s pulse duration, 2^10^ pulse frequencies, 2 mm pulse distance, and 100 cycles. Following electroporation, the *nrm*-4 cells were screened using the selective medium (SE agar plates with 30 mg/l G418). Individual clones on double selection plates were observed after 25–30 days, and they were continuously selected once per month. The individual clones grown on the selective medium were subcultured in SE liquid medium with 15 mg/L G418 and 0.25 g/l NaNO_3_.

### Identification of Transgenic Cells by Polymerase Chain Reaction (PCR)

Genomic DNA was isolated from clones grown in the selective medium with G418 using CTAB method [26]. The DNA was used for PCR analysis to detect the integration of *Np*tII gene using primer 1 and 2 (Table 1) and the Ubi-mNP-1-nos fragment using primer 3 and 4 (Table 1). Non-transgenic *nrm*-4 cells were used as a negative control, and the plasmid pGreen0029-NR-Ubi-mNP1-Nos was employed as a positive control for PCR identification. The 25 µl final reaction volume used for PCR contained 2.5 mM MgCl_2_, 250 µM each dNTP, 10 pM each primer and 2.5 units thermo-stable DNA polymerase. The PCR conditions were as follows: 95°C for 5 min and then 95°C for 1 min, 58°C for 1 min and 72°C for 2 min for 35 cycles, which was followed by a 10-min incubation at 72°C. The PCR products were analyzed by electrophoresis on 1.0% (w/v) agarose gels and by sequencing.

**Table 1:** Primers used in this study.

Primers	Sequences
Primer 1	5′ GTCCTGATAGCGGTCCGCCAC 3′
Primer 2	5′ TCCGGTGCCCTGAATGAACT 3′
Primer 3	5′ GGAACTGTATGTGTGTGTCATAC 3′
Primer 4	5′ TATGATAATCATCGCAAGACC 3′

### Extraction and Purification of mNP-1

The mNP-1 transgenic *nrm*-4 cells were cultured in 500 ml of SE solution containing 15 mg/l G418 in a rotary shaker (160 rpm) at 22°C under illumination at 156 µmol/m2/s with a 16 h light/8 h dark cycle. After 14 days, these cells were harvested by centrifugation and the pellets were re-suspended in 3-fold 25 mM NaAc/HAc buffer (pH 5.2). The cells were then disrupted for 35 s using high-pressure cell disrupter (JG-1A, Xinzhi Biotechnology Co., Ltd. Ningbo, China) and incubated at 4°C overnight. The mixture was then centrifuged, and the supernatant was used to purify mNP-1 as described previously [11], with some modification. Briefly, the soluble material was dialyzed exhaustively against 0.1% acetic acid in D-tubing (Merck Sharp & Dohme Corp.) with a molecular weight cutoff of 3,500 Dalton, lyophilized, and dissolved at 10 ml 1.0% acetic acid containing 3.0 M urea. Samples were chromatographed on a 2.5 ×120-cm column of G-25 (Pharmacia & Upjohn) that had previously equilibrated with 1.0% acetic acid. The elution peak fractions were collected at a flow rate of 20 ml/h while monitoring at A_280._ All fractions from elution peaks were analysed for the activity against *E. coli*. Following 4 continuous chromatographed with G-25, the fraction with the highest activity was subjected to Tricine-SDS-polyacrylamide gel electrophoresis (Tricine-SDS-PAGE). Further purification and analysis of the purity were performed using reversed-phase high-pressure liquid chromatography (RP-HPLC) on UltiMate 3000 (Dionex, Sunnyvale, USA) using a large-pore (300 Å) Ultimate**®**XB-C18 column (0.46×25 cm, Welch Materials, Inc., Shanghai, China). A line Water-acetonitrile gradients containing 0.1% trifluoro-acetic acid (TFA) were used for elution. The RP-HPLC analysis parameters were as follows: monitor wavelength, 280 nm; Buffer A, 0.1% TFA in water; Buffer B, 0.1% TFA in Acetonitrile; Gradient (linear), 0%–100%; buffer B in 0–50 min; Flow rate, 1 ml/min.

### MALDI-TOF-MS Analysis and Sequence Determination

The mNP-1 that was isolated from RP-HPLC was analyzed using MALDI-TOF-MS on Bruker Autoflex (Bruker, Germany). N-terminal sequencing was performed with automated Edman degradation on an Applied Biosystems procise491 instrument (Applied Biosystems, USA) by the Peking University protein analysis center. The peptide was sequenced directly without enzyme digestion or reduction of disulfide bonds.

### Anti-microbial Assay

The minimum bactericidal concentrations (MBCs) of purified mNP-1 against *E. coli* ATCC25922 (China General Microbiological Culture Collection Center) were identified using bacteria grown in liquid LB (Luria Bertani) medium by multiple double-dilute methods using initial concentration of 80 µg/ml mNP-1 [30] and Ampicillin (Amp, cellgro, Mediatech Inc., Virginia Herndon, USA) as positive control.

### Comparative Analysis of Anti-microbial Activity between mNP-1 and Amp

*E. coli* ATCC 25922 (at a concentration of 1.4 ×10^10^ CFU/ml) were mixed with increasing concentrations of mNP-1and Amp (concentration gradient: 0, 0.25, 0.5, 0.75, 1, 5, 10, 20, 40 and 50 µg/ml) respectively, and incubated at 37°C for 30 min. Surviving *E. coli* ATCC 25922 were counted using colony forming units (CFU) on plates. Bactericidal activity was represented as percentage survival versus concentration of factors (i. e. CFU/ml) in the presence of mNP-1 and Amp.

### Stability Detection and Expanding Cultivation

Ten transgenic strains, which were screened 5 times on SE solid medium containing 30 mg/l G418, were used to monitor genetic stability and mNP-1 expression. These strains were cultured in a flask containing 300 ml Endo solution [31] in a rotary shaker (160 rpm) at 22°C under illumination (156 µmol/m^2^/s) with a 16 h light/8 h dark cycle. After 14 days, culture mixture from each of the 10 transgenic strains was divided into three separate portions. The first portion was used to isolate genomic DNA and was analyze by PCR. The second portion was used to extract total proteins to test mNP-1 activity against *E. coli*. The third portion was subcultured in another flask containing 300 ml Endo solution [31] in a rotary shaker (160 rpm) at 22°C under illumination (156 µmol/m^2^/s) with a 16 h light/8 h dark cycle. We continued this protocol for each of the 10 transgenic strains for 15 consecutive cycles. DNA extraction and PCR detection are performed as described above. The total soluble proteins were extracted and used to test mNP-1 activity against *E*. *coli* and were extracted as followings: cells were harvested by centrifugation and these pellets were suspended using 25 mM NaAc/HAc buffer (pH 5.2) in the proportion of 1 g wet weight/1 ml 25 mM NaAc/HAc buffer (pH 5.2). These mixtures were disrupted for 35 s by high-pressure cell disrupter (JG-1A, Xinzhi Biotechnology Co., Ltd. Ningbo, China) and incubated at 4°C overnight. The disrupted mixtures were then centrifuged and the supernatants were used to detect the anti-microbial activity against *E. coli* ATCC25922, which was grown in LB liquid medium with double multiple dilute methods [30]. The total soluble proteins of non-transgenic *nrm-*4, isolated using the same protocol, were employed as a negative control for anti-microbial activity. Three of ten strains were selected for enlargement (up to 20 liters), using 1 volume algae cultures/4 volume Endo solution [31] with 12 of 3 l flasks on a low-speed shaker for 14 days. The anti-microbial activities of mNP-1 against *E. coli* ATCC25922 were detected using multiple double-dilute methods [30].

### Time-course Cell Growth and Anti-microbial Activity Detection of Transgenic Cells

The transgenic strain 6-2 and non-transgenic *nrm*-4 were cultured as an initial inoculation quantity about 3 g wet cell weight per liter in a flask containing 500 ml Endo solution [31] in a rotary shaker (160 rpm) at 22°C under illumination (156 µmol/m^2^/s) with a 16 h light/8 h dark cycle. The biomass (wet weight) was tested every day. The anti-microbial activity of transgenic strains against E. coli ATCC25922 was detected during the time-course cell growth using multiple double-dilute methods.

## Results

### Construction of NP-1 Plant Expression Vector and Transformation of nrm-4

In this study, we used double-selection marker genes (*NR* and *Npt*II) to improve the selection efficiency of transformed cells. The final construct is shown in Fig. 1A. We used electroporation method to transform the NR-deficient *C. ellipsoidea* mutant *nrm*-4 cells, the transformed *nrm*-4 cells were screened on double selection medium i.e. SE agar plates containing 30 mg/l G418 and 0.25 g/l NaNO_3_. Hundreds of individual clones could be observed 25–30 days after transformation (Fig. S1). The overall efficiency was estimated to be approximately 750 clones, per electroporation, with G418 and NaNO_3_ resistance. Untransformed *nrm*-4 cells can’t grow on above medium. To maintain the stability of the transgenic *nrm*-4, the individual clones growing on the selective medium were separately diluted in a 50 µl dilution tube containing SE solution and spread on double selection plates once a month. This process is called purification of transgenic strains. To obtain stable transgenic lines, at least 5 times of continuous selection were performed.

### Identification of Transgenic nrm-4 by PCR

The clones growing on the double selection medium for 25–30 days after transformation were subcultured in SE solution containing 15 mg/l G418 and 0.25 g/l NaNO_3_ for 4 days and then used to detect the part of *Npt*II gene (501 bp) with primers 1 and 2 (Table 1), and a part of Ubi-mNP1-Nos fragment (552 bp) with primers 3 and 4 (Table 1). *Npt*II gene and Ubi-mNP1-Nos fragment were detectable in 80% of clones initially growing on double selection plates, and 100% after 3 cycle selections. Fig. 1B and Fig. 1C were representative results for detection of *Npt*II gene and Ubi-mNP1-Nos fragment, respectively. The PCR products were confirmed further by sequencing analysis. Thus, we considered the plasmid pGreen0029-NR-Ubi-mNP1-Nos had been integrated into the genome of *nrm*-4.

### Purification and Characterization of mNP-1

**Figure 2:**
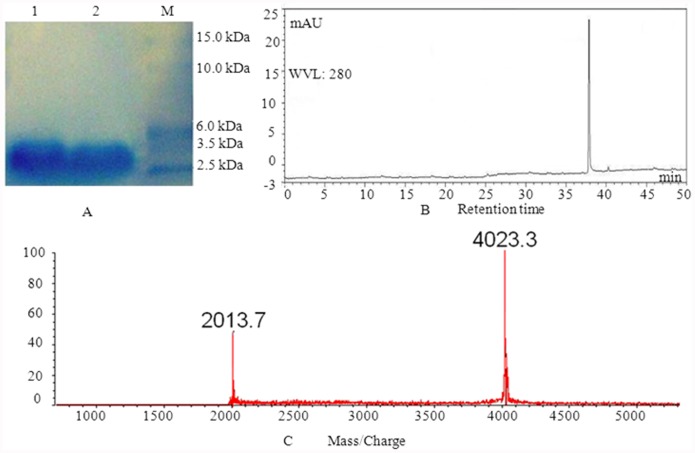
Characterisation of the purified mNP-1. (**A**) Tricine-SDS-polyacrylamide gel electrophoresis (Tricine-SDS-PAGE) of purified mNP-1. Extracted proteins from transgenic *nrm*-4 cells were dialysed with a molecular weight cut-off of 3,500 Da and chromatographed using a G-25 column (Pharmacia & Upjohn, NJ, USA). The fractions with the activity against *E. coli* were collected and continuously chromatographed 4 times. Purified peptides (15 µg per lane) from the G-25 chromatography were analysed using Tricine-SDS-PAGE (10–20% gel gradient). The mNP-1 migrates faster than a similarly sized molecular weight marker (M) due to its greater charge. mNP-1 denoted by 1 and 2. (**B**) Reversed-phase high-pressure liquid chromatography (RP-HPLC) of purified mNP-1. A mixture containing 20 µg each of mNP-1 was applied to a C-18 column and developed using a linear water-acetonitrile gradient at a flow rate of 1 ml/min and monitored at 280 nm on a UltiMate 3000 (Dionex, Sunnyvale, USA). (**C**) MALDI-TOF-MS analysis of purified mNP-1. The mNP-1 isolated from RP-HPLC was analysed by MALDI-TOF-MS using a Bruker Autoflex (Bruker, Germany). The peak at the molecular weight, 4023.3 Da, is the mNP-1 with a single charge; another peak of molecular weight 2013.7 Da is the mNP-1 containing two charges.

Extracts from transgenic lines were purified with size exclusion column G-25. Elution peaks were collected by division. All fractions from elution speaks were tested the activity against *E*. *coli* ATCC25922. The fractions with highest activity from continuously chromatographed for 4 times by G-25 were analyzed by Tricine-SDS-PAGE. A prominent band could be observed (Fig. 2A). These samples were analyzed further by RP-HPLC (Fig. 2B). The mNP-1 products from RP-HPLC were collected and concentrated. Peptide preparations generated by RP-HPLC was detected by MALDI-TOF-MS, whose mass-to-charge ratio (Fig. 2C) was consistent with the expected value of mNP-1 (molecular weight, 4023.3 Dalton). The amino acid sequence analysis of the peptide was performed by automated Edman degradation. Fifteen amino acids of mNP-1 were read in the N-terminal sequencing, 12 of which were determined (Table 2). The N-terminal Met was still retained in the mNP-1 expressed by *Chlorella*.

**Table 2:** N-terminal sequence of mNP-1 determined by automated Edman degradation.

No.	1	2	3	4	5	6	7	8	9	10	11	12	13	14	15
AA	Met	Val	Val	–	Ala	–	Arg	Arg	Ala	Leu	–	Leu	Pro	Arg	Glu

Note: In theory, cysteines exist at the 4^th^, 6^th^ and 11^th^ positions, and we observed no absorption peaks here due to the formation of disulphide bonds.“AA” denotes amino acid; “−” indicates no absorption peak.

### The Detection of mNP-1 Antimicrobial Activity

**Figure 3:**
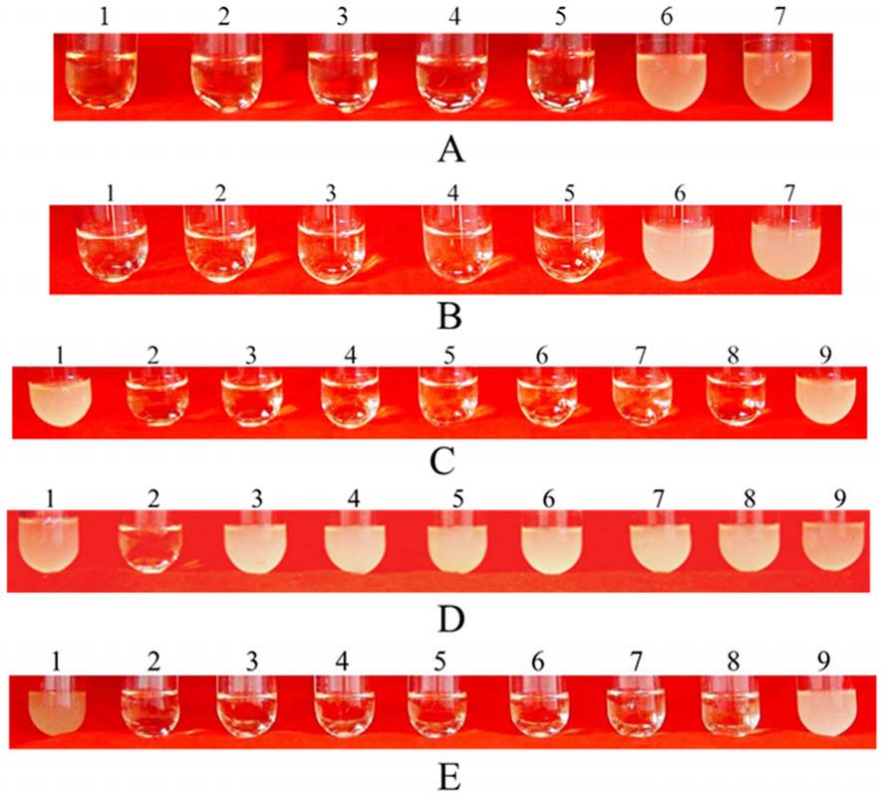
The detection of mNP-1 anti-microbial activity against *E. coli.* ( A) The MBC determination of Amp against *E. coli* ATCC25922. The 800 µg/ml Amp was used as initial concentration to detect the MBC against *E. coli* ATCC25922 by double multiple dilute methods using liquid LB medium. The concentration of bacteria incubated in this assay is 1 ×10^6^ CFU/ml. (1–6) 800, 400, 200, 100, 50 and 25 µg/ml Amp; (7) Negative control (without any Amp). (B) The MBC of mNP-1 against *E. coli* ATCC25922. An initial concentration of 80 µg/ml mNP-1 (obtained from RP-HPLC) was used to detect MBC against *E. coli* ATCC25922, and this solution was diluted multiple times by double-dilution methods using liquid LB medium. The concentration of bacteria used for this assay was 1 ×10^6^ CFU/ml. (1–6) 80, 40, 20, 10, 5 and 2.5 µg/ml mNP-1; (7) negative control (without any mNP-1). (C–E) Antimicrobial activity of total soluble protein extracted from transgenic *nrm*-4 cells. Total soluble protein extracted from cultures of transgenic *nrm*-4 cells without G418 selection stress **(**C), non-transgenic *nrm*-4 cells (D) and enlarged cultures of transgenic *nrm*-4 without G418 selection stress (E). (1) bacterial control; (2) negative control (LB liquid medium); (3–9) total soluble proteins diluted 2-, 4-, 8-, 16-, 32-, 64- and 128-fold with LB liquid medium, respectively.

The anti-microbial activity against *E*. *coli* ATCC25922 was detected by double multiple dilute methods [30] using 80 µg/ml of mNP-1 from RP-HPLC as initial concentration (Fig. 3B). As a positive control, 800 µg/ml of Amp as initial concentration were used to test the anti-microbial activity against *E*. *coli* ATCC25922 (Fig. 3A). The 100 µl mixtures of tube 5 in Fig. 3A and B were spread on LB agar plates and incubated at 37°C overnight. The average number of clones per plate was less than 5, which indicated that 99.9% of bacteria were killed in the culture mixtures. Therefore, the MBCs of mNP-1 and Amp against *E. coli* were 5 µg/ml and 50 µg/ml, respectively.

**Figure 4:**
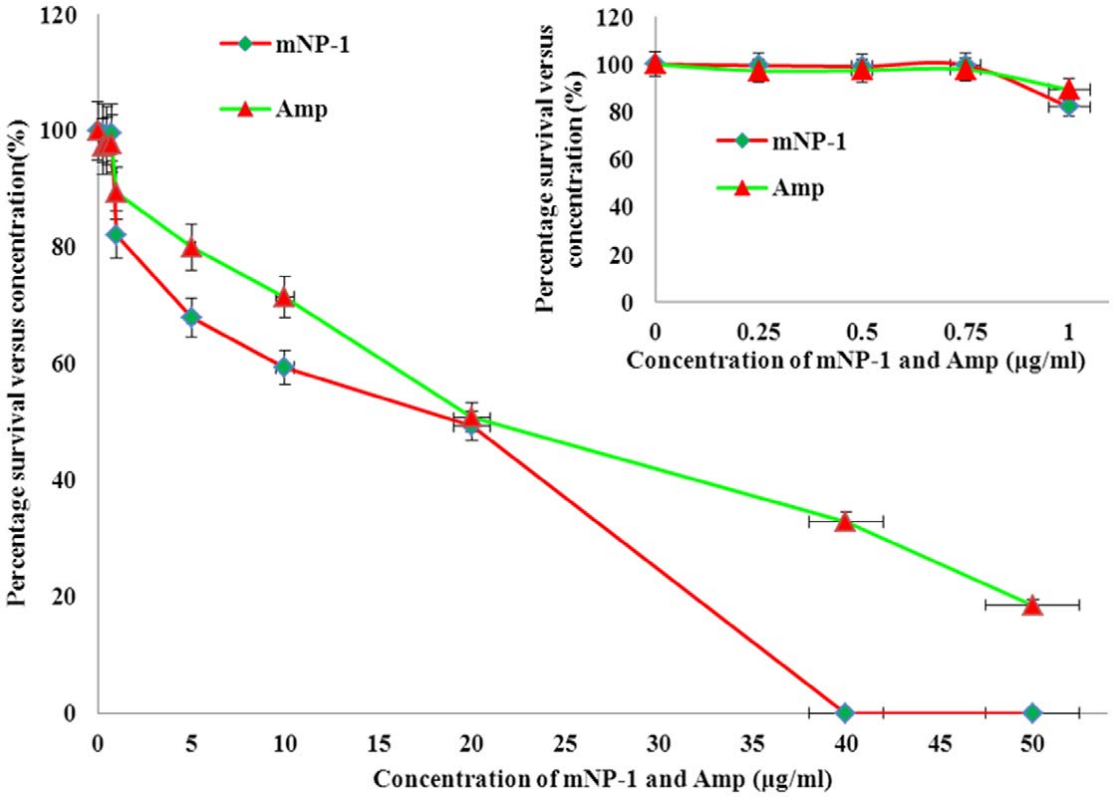
Comparative analysis of mNP-1 and Amp against *E. coli* ATCC 25922. *E. coli* ATCC 25922 (at a concentration of 1.4 ×10^10^ CFU/ml) was mixed with increasing concentrations of mNP-1 and Amp (concentration gradient: 0, 0.25, 0.5, 0.75, 1, 5, 10, 20, 40 and 50 µg/ml), respectively, and incubated at 37°C for 30 min. Surviving *E. coli* ATCC 25922 were counted using colony forming units (CFU) on plates. Results of bactericidal activity were represented as percentage survival versus concentration (i.e. CFU/ml) in the presence of mNP-1 and Amp. Upright figure is an enlarged part of Figure 4 at concentration of 0–1 µg/ml.

In addition, we compared the bacterial killing activity between mNP-1and Amp with increasing concentrations (concentration gradient: 0, 0.25, 0.5, 0.75, 1, 5, 10, 20, 40 and 50 µg/ml). Results of bactericidal activity were represented as percentage survival versus concentration (i. e. CFU/ml) in the presence of mNP-1 and Amp (Fig. 4). The bactericidal activities of mNP-1 and Amp were similar and weak at the lower concentration, e. g. 0–0.75 µg/ml; the anti-microbial activities of mNP-1 were significantly stronger than that of Amp at concentration of 1–10 µg/ml, and 40–50 µg/ml, when they were incubated with *E. coli* (a concentration of 1.4 ×10^10^ CFU/ml) at 37°C for 30 min. The bactericidal activity of mNP-1 against *E. coli* was similar to that of Amp at the concentration of 20 µg/ml.

### Transgenic Strain Stability and Cultivation Expansion

Ten of transgenic strains screened on solid selection medium for 5 times were continuously subcultured in liquid medium without antibiotic G418 once every two weeks for 15 times. The genetic stability was detected by PCR. PCR results indicated the *Npt*II gene and Ubi-mNP1-Nos fragments were remained in the genomes of ten transgenic strains (same as Fig. 1B and 1C, respectively). The mNP-1 production was assayed by antimicrobial activity against *E*. *coli*. The antimicrobial activity results showed that all 64-fold dilutions of total soluble protein from these strains can kill *E*. *coli* ATCC25922 (Fig. 3C). As negative control, the total soluble protein from non-transgenic *nrm*-4 has no activity against *E. coli* ATCC25922 (Fig. 3D).

Cultures of three strains were separately increased to 20 l with 12 of 3 l flasks on low-speed shaker, and were cultured for 14 days. The average biomass is up to 35.68±5.41 g wet weight/l, and their total soluble proteins diluted 64-fold could also kill *E. coli* ATCC25922 (Fig. 3E). Since the MBC of mNP-1 was 5 µg/ml, it was estimated that the production of mNP-1 was at approximately 11.42 mg/l.

We detected the time course growth of transgenic cells and non-transgenic cells *nrm*-4. The cell grow at the lag phase within 1–2 d and 8–10 d, and at the exponential phase from the 3^th^ d to 6^th^ d, and 10^th^ d to 12^th^ d, then at stationary phase after the 13^th^ d (Fig. S2). There is no significant difference in growth between transgenic strain and non-transgenic *nrm*-4. We detected the mNP-1 efficient expression level by testing the bactericidal activity of mNP-1 during culture period and found that the efficient mNP-1 is increasing with culture time course and up to the highest level at 14^th^ day (Fig. S3). It suggested that the highest efficient expression level of mNP-1 could be at stationary phase, a suitable time to harvest mNP-1.

## Discussion

### High-efficient Transformation and Stability of Transgenic Chlorella Ellipsoidea

The algae transformation was successfully achieved using glass bead, silicon carbide whiskers, particle bombardment, and electroporation-mediated transformation methods in different species [32]. As a genus of single-cell green algae, *Chlorella* multiplies rapidly, and some of the species such as *C. pyrenoidosa*, *C. vulgaris* and *C. ellipsoidea* can serve as potential source of food and energy because of their high photosynthetic efficiency [33]. These characteristics of *Chlorella* allow the realization of its potential to generate valuable products using genetic engineering. However the research information on genetic engineering of *Chlorella* is limited. The important first transformation of *C. ellipsoidea* was the transformation of protoplasts achieved by polyethylene glycol (PEG) media in transient expression of firefly luciferase using *C. ellipsoidea* protoplasts [22]; and followed by the plasmid pCaMVCAT transient expression in *C. saccharophila* protoplast performed by electroporation [23]. The rescue transformation in NR-deficient *Chlorella sorokiniana* using *NR* gene from *C. vulgaris* was successfully established by bombardment and transformants were able to grow on selective medium [21]. It was the first to confirm that the *NR* gene could be used as a selection pressure gene. *C. vulgaris* C-27 and *C. sorokiniana* were used to express human growth hormone (HGH) by PEG-media transformation. The HGH transient expression was detectable in 6 h after transformation. A dozen of colonies were achieved in transfected 10^9^ cells and none of these colonies produced HGH [24]. *C. ellipsoidea* protoplast was used to express flounder growth hormone (FGH) gene controlled by Cauliflower Mosaic Virus (CaMV)-35 S promoter by PEG-mediated transformation and the expression amount of FGH is 400 µg/l estimated by ELISA [25].

NR-deficient *C. ellipsoidea* mutant *nrm*-4 was previously developed by our group using X-ray mutagenesis method [27]. It grew in the medium with nitrite,but not in the medium with nitrate. Therefore, *NR* gene can be used as a selective marker when *nrm*-4 was used as the host cell using nitrate as the selection chemical. *NR* gene from *C. ellipsoidea* was cloned and characterized by our group [27]. Our previous work also proved that *Npt*II gene could be used as a selective marker in the transformation of *C. ellipsoidea* using G418 as the selection chemical [26].

In this study, we established an efficient transformation method using innate cells (rather than protoplasts) as host, *NR* and *Npt*II as double selection marker genes, and simple electroporation. Approximately 750 clones with G418 and nitrate resistance could be obtained in each electroporation transformation, of which up to 80% had the foreign gene.

In our previous work, we got transgenic strains of *C. ellipsoidea* with anti-microbial activity [26], but the transgenic cells could be lost when cultured in the medium without any selection stress (unpublished data). In this study, we found that the transgenic cells were genetically stable after being selected on solid selection medium for 5 times (once per month). We guess that the transgenic strains obtained by Chen et al. (2001) were not pure transgenic strains. One reason is due to lack of the cell purifying process in Chen’s et al. study. Another reason is that in this study we used two selection marker genes that would be helpful to rapidly get pure transgenic strain under G418 and nitrate stresses. Therefore, it is necessary to purify the transgenic cells in the transgenic research on *C. ellipsoidea.*

### Biochemical Identification of mNP1

In rabbit, NP-1 is synthesized as 95 amino acid (aa) preprodefensin. The 95 aa putative precursor contains a typical 19 aa signal sequence, a 43 aa anionic propiece and a 33 aa mature peptide. The 33 aa peptide comprises the whole C-terminus of the preprodefensin that is proteolytically cleaved to release the C-terminal mature peptide and become activated NP-1 with 33 aa [34]. To express the active peptide, we added an initiator codon ATG in the 5′-end of NP-1 gene when synthesizing 33 aa gene sequence. Previous studies indicated that excision of translation initiator methionine was critical for their function and stability of recombinant proteins for human hemoglobin, interleukin-2, growth hormones, or frog ribonucleases [35]. In eukaryotes and prokaryotes, N-terminal Met excision (NME) of proteins is mainly performed by Met aminopeptidases (MAPs). MAP activity depends on the nature of the second residue in protein. If it is small side-chain amino acid, such as Gly, Ala, Pro, Cys, Ser, Thr or Val, the methionine is cleaved; otherwise it is retained [36]. But many exceptions were found in *E. coli* proteins [37]. In this study, the result of N-terminal sequencing (Table 2) showed that the N-terminal methionine of the mNP-1 expressed in *C. ellipsoidea* was still retained, although the second residue of mNP-1 is Val. Our results also indicated that the mNP-1 with N-terminal methionine had strong anti-microbial activity (Fig. 3B) and was stable during prolonged culture and through the process of purification and activity test. We provided a clue to understanding the post-translational processing of proteins from either native or recombinant proteins in *C. ellipsoidea*.

The differences of α-defensins were mainly caused by the N-and C-terminal residues and the variation of N-and C-terminal residues influenced the activity and antibacterial spectrum of α-defensins [38]. mNP-1 has just one more amino acid (i.e. Met) than NP-1 at the N-terminus of sequence, but the amphiphilics may be different from previously reported that of NP-1 [11]. In RP-HPLC, the retention time of mNP-1 and NP-1 is about 38–40 min (Fig. 2B) and 16–18 min [11], respectively. The reason needs to be further investigated. Does it have effect on the activity of mNP-1? Compared with previous report, it seems that the anti-microbial activities of mNP-1 are significantly stronger than that of NP-1. For instance, MBC of mNP-1 against *E. coli* is 5 µg/ml, but 50 µg/ml NP-1 only kill a small amount of *E. coli* [11]. In this study we did not compare the anti-microbial activity between mNP-1 and NP-1 due to lack of active NP-1 sample. However it should be performed in future work. Importantly, the comparison of the bactericidal activities of mNP-1 and Amp showed that bactericidal activities of mNP-1 was stronger than that of Amp at low concentration (1–10 µg/ml) (Fig. 4).

### The Potential of Defensin Production by Chlorella

Using higher plants as bioreactors to produce biopharmaceuticals has achieved great advances [39], especially those by chloroplast engineering due to higher production of foreign proteins and maternal inheritance of transgenes [40]. In single cell plants, some therapeutic proteins also successfully expressed [41]–[43]. Compared with higher plants, the production of *Chlorella* can be easily controlled. Compared with other eukaryotic single cell plants, such as *Chlamydomonas reinhardtii*, *Chlorella* grows much faster. *Chlorella* has high growth rate and can be used as feedstocks, agriculture and health products. Under heterotrophic growth conditions, *Chlorella* biomass yields can reach cell densities of 50–100 g of dry biomass per liter, and is comparable to the 130 g/l of yeast dry biomass of commercial fermenter [44], lower than the 210 g/l of *E*. *coli* dry biomass of dialysis fermenters [45]. Our results showed that the cell growth of transgenic lines was similar to that of non-transgenic *nrm*-4 (Fig. S2). Therefore, *Chlorella* has a potential to be a new host cell system for large-scale production of biopharmaceuticals in biotechnology.

Due to the characteristics of defensins in anti-microbial activity, only limited success has been achieved in defensin production by genetic engineering, although different defensins had been successfully expressed in *E. coli* by fusion protein strategy, and in yeast by secretion [8], and HBD1 [9]. In our study, mNP-1 output is up to 11.42 mg/l; its activity against *E*. *coli* ATCC25922 is strong; the transgenic lines is genetically stable and mNP-1 gene from rabbit can be expressed directly without codon modification. Therefore, Chlorella expression system could be a competitive system for production of defensins. In addition, Chlorella expression system can be also used as bioreactors to produce other biologically active substances. However, a large scale production of defensins needs to be evaluated by photoautotrophic/heterotrophic, which is currently undertaken by our group.

### Conclusion

In conclusion, we developed a new strategy to produce defensins in *C. ellipsoidea*. The mNP-1 can be produced at a high level in *C. ellipsoidea* with strong anti-microbial activity. Our results suggested that *C. ellipsoidea* should have a potential to be used as a new bioreactor to produce defensins as well as other valuable bioactive compounds.

## Supporting Information

Figure S1 Transgenic colony screening.
(DOC)

Figure S2 The cell growth curve of transgenic strain 6-2 and non transgenic cell *nrm*-4.
(DOCX)

Figure S3 The anti-microbial activity of transgenic strains against *E. coli* ATCC25922 during the time-course cell growth.
(DOC)
