# Supplementary material for: A New Strategy to Produce a Defensin: Stable Production of Mutated NP-1 in Nitrate Reductase-Deficient Chlorella ellipsoidea
Source: PLoS One. 2013 Jan 28;8(1):e54966. doi: 10.1371/journal.pone.0054966 (PMC3557228; doi:10.1371/journal.pone.0054966)
Supplement: Figure S1 — Transgenic colony screening. (DOC) [file pone.0054966.s001.doc]

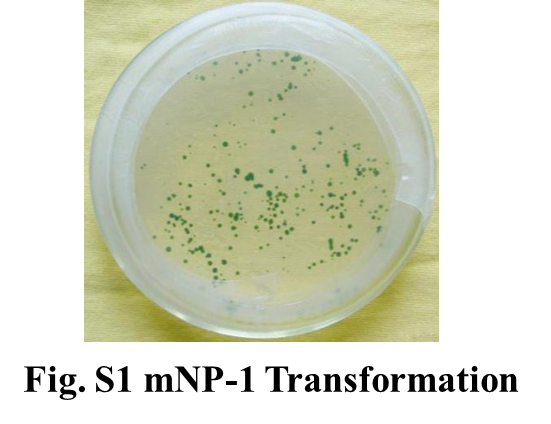


**Fig. S1 Transgenic colony screening.** G418-resistant clones of *Chlorella ellipsoidea* *nrm*-4 on double-selection plates containing 30 mg/l G418 (Geneticin) and 0.25 g/l sodium nitrate for 30 days after electroporation of the two plasmids pSoup (Biotechnology and Biological Sciences Research Council (BBSRC), UK) and pGreen0029-NR-Ubi-mNP1-Nos.
