# Supplementary material for: A New Strategy to Produce a Defensin: Stable Production of Mutated NP-1 in Nitrate Reductase-Deficient Chlorella ellipsoidea
Source: PLoS One. 2013 Jan 28;8(1):e54966. doi: 10.1371/journal.pone.0054966 (PMC3557228; doi:10.1371/journal.pone.0054966)
Supplement: Figure S2 — The cell growth curve of transgenic strain 6-2 and non transgenic cell nrm-4. (DOCX) [file pone.0054966.s002.docx]

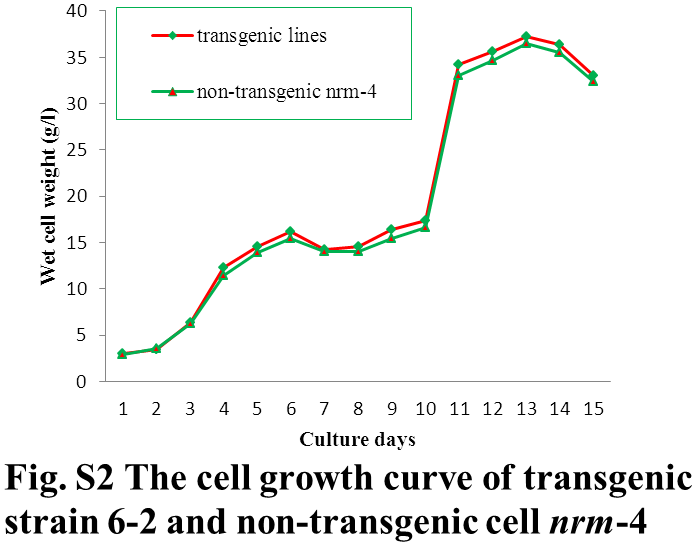


**Fig. S2 The cell growth curve of transgenic strain 6-2 and non transgenic cell *nrm*-4**

The transgenic strain 6-2 and non transgenic cell nrm-4 were cultured in a flask containing 500 ml Endo solution in a rotary shaker (160 rpm) at 22°C under illumination (156 μmol/m^2^/s) with a 16 h light/8 h dark cycle. The biomass (wet weight) was tested every day.
