# Supplementary material for: A New Strategy to Produce a Defensin: Stable Production of Mutated NP-1 in Nitrate Reductase-Deficient Chlorella ellipsoidea
Source: PLoS One. 2013 Jan 28;8(1):e54966. doi: 10.1371/journal.pone.0054966 (PMC3557228; doi:10.1371/journal.pone.0054966)
Supplement: Figure S3 — The anti-microbial activity of transgenic strains against E. coli ATCC25922 during the time-course cell growth. (DOC) [file pone.0054966.s003.doc]

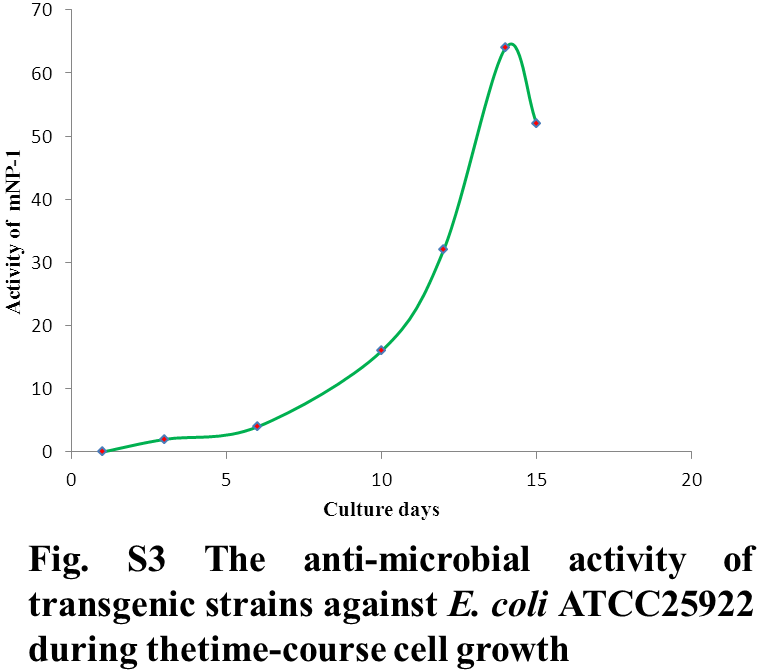


**Fig. S3 The anti-microbial activity of transgenic strains against *E. coli* ATCC25922 during the time-course cell growth**

The ordinate represented anti-microbial activity of the total soluble protein from transgenic lines during time-course cell growth by double multiple dilute methods using liquid LB medium. The number was the maximum diluted times of the total soluble protein killing the *E. coli* ATCC25922 (a concentration of 1 ×10^6^ CFU/ml).
